# Early exposure to social disadvantages and later life body mass index beyond genetic predisposition in three generations of Finnish birth cohorts

**DOI:** 10.1186/s12889-020-08763-w

**Published:** 2020-05-18

**Authors:** Estelle Lowry, Nina Rautio, Niko Wasenius, Tom A. Bond, Jari Lahti, Ioanna Tzoulaki, Abbas Dehghan, Anni Heiskala, Leena Ala-Mursula, Jouko Miettunen, Johan Eriksson, Marjo-Riitta Järvelin, Sylvain Sebert

**Affiliations:** 1 Center for Life Course Health Research, University of Oulu, P.O.Box 5000, Fin-90014, Oulu, Finland; 2 Biocenter Oulu, University of Oulu, P.O.Box 8000, Fin-90014, Oulu, Finland; 3 School of Natural and Built Environment, Queen’s University Belfast, Elmwood Avenue Belfast, Belfast, BT7 1NN UK; 4 Unit of Primary Health Care, Oulu University Hospital, P.O.Box 10, 90029 OYS Oulu, Finland; 5 Folkhälsan Research Center, Haartmanninkatu 8, 00290 Helsinki, Finland; 6 Department of General Practice and Primary Health Care, University of Helsinki, P.O. Box 20, 00014, Helsinki, Finland; 7 Department of Epidemiology and Biostatistics, Imperial College, London, SW7 2AZ UK; 8 Department of Psychology and Logopedics, University of Helsinki, P.O.Box 63, 00014, Helsinki, Finland; 9 Turku Institute for Advanced Studies, University of Turku, FI-20014 Turku, Finland; 10 Department of Hygiene and Epidemiology, University of Ioannina Medical School, 45110 Ioannina, Greece; 11 Department of Biostatistics and Epidemiology, MRC-PHE Centre for Environment and Health, School of Public Health, Imperial College London, London, W2 1PG UK; 12 Medical Research Center Oulu, Oulu University Hospital and University of Oulu, P.O. Box 8000, FI-90014 Oulu, Finland; 13 Department of Obstetrics and Gynecology, National University of Singapore, Yong Loo Lin School of Medicine, Singapore, SG Singapore; 14 Singapore Institute for Clinical Sciences, Agency for Science, Technology, and Research, Singapore, Singapore; 15 Department of Genomics, Imperial College London, London, SW7 2AZ UK

**Keywords:** Early life, Social disadvantage, Body mass index (BMI), Maternal, Polygenic risk score for BMI

## Abstract

**Background:**

The study aimed to explore the association between early life and life-course exposure to social disadvantage and later life body mass index (BMI) accounting for genetic predisposition and maternal BMI.

**Methods:**

We studied participants of Helsinki Birth Cohort Study born in 1934–1944 (HBCS1934–1944, *n* = 1277) and Northern Finland Birth Cohorts born in 1966 and 1986 (NFBC1966, *n* = 5807, NFBC1986, *n* = 6717). Factor analysis produced scores of social disadvantage based on social and economic elements in early life and adulthood/over the life course, and was categorized as high, intermediate and low. BMI was measured at 62 years in HBCS1934–1944, at 46 years in NFBC1966 and at 16 years in NFBC1986. Multivariable linear regression analysis was used to explore associations between social disadvantages and BMI after adjustments for polygenic risk score for BMI (PRS BMI), maternal BMI and sex.

**Results:**

The association between exposure to high early social disadvantage and increased later life BMI persisted after adjustments (β = 0.79, 95% CI, 0.33, 1.25, *p* < 0.001) in NFBC1966. In NFBC1986 this association was attenuated by PRS BMI (*p* = 0.181), and in HBCS1934–1944 there was no association between high early social disadvantage and increased later life BMI (β 0.22, 95% CI –0.91,1.35, *p* = 0.700). In HBCS1934–1944 and NFBC1966, participants who had reduced their exposure to social disadvantage during the life-course had lower later life BMI than those who had increased their exposure (β − 1.34, [− 2.37,-0.31], *p* = 0.011; β − 0.46, [− 0.89,-0.03], *p* = 0.038, respectively).

**Conclusions:**

High social disadvantage in early life appears to be associated with higher BMI in later life. Reducing exposure to social disadvantage during the life-course may be a potential pathway for obesity reduction.

## Background

Obesity continues to be a major threat to public health resources [1]. The Global epidemic of obesity is driven by shared societal determinants and appear to have the greatest effect on the poorest people [2]. Current economic systems are promoting excessive and unsustainable consumption patterns, which are reflected in the rising obesity levels worldwide [2]. Therefore, there is an urgency to address the continuing challenge of obesity in terms of biological and social risks in early life. In simplest terms, it may seem as though the rising obesity prevalence is due to environmental exposures such as excessive energy intake, sedentary lifestyles, [3] and sleep debt [4]. However, it appears to manifest preferentially in genetically predisposed individuals [5] suggesting a more complex interaction between hereditary/genetic and environmental risk factors [6].

Accumulating evidence supports an important role for social factors in early life that may be equally, if not more important than adult social factors in predisposing to adult obesity [7–10]. A previous study of Northern Finland Birth Cohort 1966 showed that differences in BMI by social class were formed at least partly during early childhood and a high maternal pre-pregnancy BMI was predictive of obesity in adulthood among the offspring [11]. Since then, early social disadvantage has been continually linked to later obesity [7–9]. One study also reported that higher genetic risk and low socio-economic status were associated with higher BMI in pre-adolescent individuals [12]. We hypothesised that a higher genetic risk for obesity may already be present at birth in the most socially disadvantaged groups (Additional File 1, Fig. 1). However, it is still unclear whether the association between early social disadvantage and later life obesity is direct or due in part to the inheritance of genetic risk and/or other maternal risk factors. Further complicating this relationship is the concept of social mobility, in which people can move up or down the social hierarchy throughout the life-course. Previous research has shown the direction of mobility to be accompanied by improvement or worsening of health, and likewise changes in health can often be related to fluctuations in social circumstances [13, 14]. Therefore, more information is needed on whether the association between early social disadvantage and later obesity can be modified by the process of social mobility during the life-course [13].

In this study, we aimed to test whether i) early exposure to social disadvantage is related to later life body mass index (BMI) and other measures of body composition, ii) the relationship between early exposure to social disadvantage and later life BMI and body composition can be explained by higher genetic predisposition or maternal BMI and iii) reduction in social disadvantage during the life-course is associated with a lower BMI and other measures of adiposity in adulthood. Finally, we used three generations of birth cohorts in Finland to replicate the study during different historical phases of the obesity epidemic.

## Methods

The study population comprised participants from the Helsinki Birth Cohort Study (HBCS1934–1944) [15] and Northern Finland Birth Cohorts 1966 (NFBC1966) and 1986 (NFBC1986), [16], which are population-based birth cohorts.

**Fig. 1:**
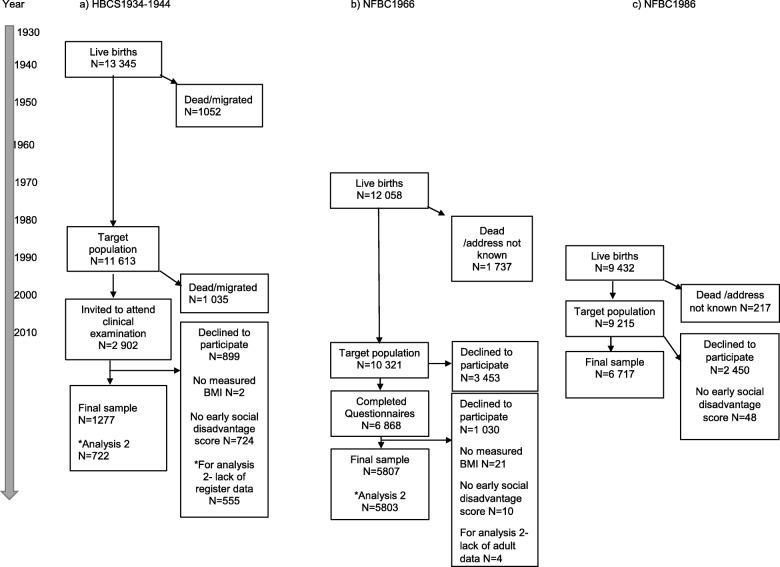
Flow chart of the three Finnish birth cohorts **a** Helsinki Birth Cohort Study (HBCS1934–1944), **b** Northern Finland Birth Cohort 1966 (NFBC1966) and **c** Northern Finland Birth Cohort 1986 (NFBC1986)

HBCS1934–1944 comprised a total of 13,345 live-born children between 1934 and 1944 at Helsinki University Central Hospital or the Helsinki City Maternity Hospital and they were identified and followed through to present day using register data [17]. Children attended child welfare clinics voluntarily and those, who were alive and living in Finland in 1971, received a unique personal identity number (ID). Information on pregnancy and early life was gathered from hospital records and child welfare clinics. ID numbers were used to link the data to national registers. Clinical examinations were conducted on a random sample of 2003 individuals, at a mean age of 62 years between 2001 and 2004, and for 2001 of those individuals, the primary outcome, i.e. measured BMI was available. The present study used the 1277 individuals with measured BMI and early life social disadvantage score in the analysis examining early life exposure to social disadvantage with adulthood BMI. In the second analysis examining social mobility, a sample size of 722 participants had both adult register and clinical data (Fig. 1). The clinical study protocol was approved by the Ethics Committee of Epidemiology and Public Health of the Hospital District of Helsinki and Uusimaa.

NFBC1966 included 12,058 live-born children (96.3% of all births) during 1966 in the two former northern provinces of Finland, Oulu and Lapland [18]. The current analysis focuses on information provided during pregnancy and the latest 46-year follow-up, which was conducted between April 2012 and February 2014. Of the 10,321 eligible individuals at age 46-years, 5817 individuals had the primary outcome measure, i.e. measured BMI and the present study used the 5807 individuals with information concerning early life exposure and BMI (Fig. 1).

NFBC1986 consisted of 9432 live-born children between 1st of July 1985 and the 30th of June 1986 also in the former provinces of Oulu and Lapland (98.5% of all births) [16]. The data used in this study was collected during pregnancy and at age 16 years by clinical examination. Of 9215 eligible individuals at age 16 years, 6765 individuals had our primary outcome measure, measured BMI, in 2001. Overall, 6717 individuals had information concerning early life social disadvantage and BMI (Fig. 1). NFBC1966 and NFBC1986 were approved by the Ethics Committee of the Northern Ostrobothnia Hospital District.

### Social disadvantage measures used in factor scores

Parental data were used to quantify individuals’ early life exposure to social disadvantage. In HBCS1934–1944, information was obtained from hospital records, child welfare clinics and school health care records. All social disadvantage measures at mean age 44 years (range 41–51 years) in 1985 were based on national registers from Statistics Finland. In NFBCs, information concerning parental social disadvantage was obtained from questionnaires during pregnancy. At 46 years, social disadvantage measures in NFBC1966 were taken from postal questionnaire, only occupation was obtained from the national register received from Statistics Finland in 2013. In NFBC1986 social disadvantage scores were not computed at 16-year follow-up, as these would still have been reflective of the parent’s situation.

In order to create a composite measure of social disadvantage during early life and adulthood, we used a systematic approach for variable selection as described previously in [19]. Following an inventory of available variables in each dataset, we selected all those, which were

- an indicator of social disadvantage (a lack of social and economic resources) based on previous literature and a priori knowledge.
- associated with the primary outcome of later life body mass index (BMI) (*p* < 0.05).

The same steps were followed to create a social disadvantage measure in early life and adulthood within HBCS1934–1944 and NFBC1966, and in NFBC1986 during early life only as adult follow-up data is not yet available.

All available indicators of social disadvantage (Criteria 1) are listed in the Additional file 1, Tables 1A-C. The final selected variables for the composite score (meeting Criteria 2) are as follows; In HBCS1934–1944 early life social disadvantage was represented by paternal and maternal occupation and number of people per room. In adulthood,variables were higher education, household income and occupation. In NFBC1966 early life social disadvantage meeting all criteria was represented by parental marital status, paternal occupation, maternal occupation, maternal education and material wealth. Material wealth was a constructed variable including apartment/house ownership, car ownership and whether the family’s dwelling had electricity, telephone, running water and television. Adulthood social disadvantage variables were basic education, higher education, occupation, employment status and home ownership. In NFBC1986 early life social disadvantage was represented by paternal occupation, maternal occupation, maternal education and material wealth. Material wealth was a constructed variable including ownership of apartment/house, summer cottage, car, automatic washing machine, telephone, central heating, flushing toilet and a separate bathroom. Full information on original categorisations and re-categorizations are presented in Additional file 1, Table 2.

**Fig. 2:**
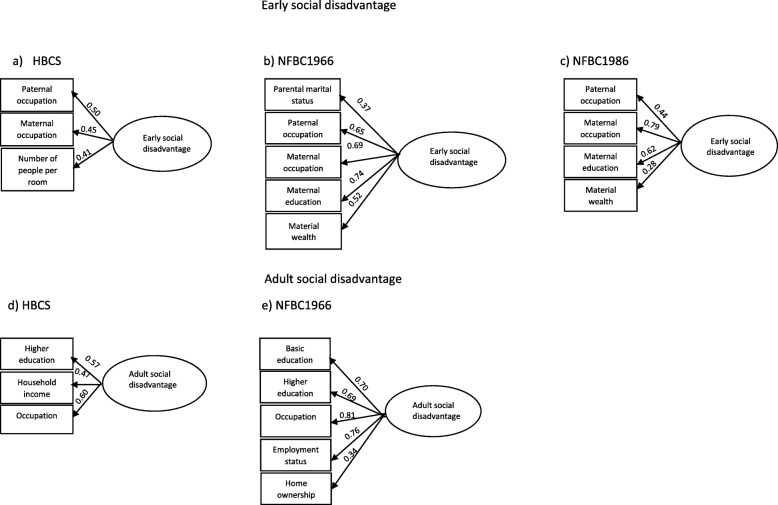
Early social disadvantage in **a** Helsinki Birth Cohort Study (HBCS1934–1944), **b** Northern Finland Birth Cohort 1966 (NFBC1966) and **c** Northern Finland Birth Cohort 1986 (NFBC1986) and adult social disadvantage in **d** HBCS1934–1944 and **e** NFBC1966

Following variable selections (Additional file 1, Tables 1A-C**)**, confirmatory factor analysis (CFA) was used to produce a single factor score to reflect social disadvantage during the early life in all three cohorts and at a point in adulthood in HBCS1934–1944 and NFBC1966 (Fig. 2). The scores were divided into quartiles with the highest 25% representing high social disadvantage, and the lowest 25% representing low social disadvantage. Participants were defined as having reduced social disadvantage in NFBC1966 and HBCS1934–1944 if they moved to a lower social disadvantage category between the two time points, stable if there was no movement and increased social disadvantage if they moved to a higher social disadvantage category.

### Body mass index and other measures of body composition

At approximately 62 years of age in HBCS1934–1944, 46 years in NFBC1966 and 16 years in NFBC1986 individuals were invited to clinical examinations conducted by trained research nurses. Weight (kg) and height (cm) were measured from participants in light indoor clothing without shoes. BMI (kg/m^2^) was calculated as weight (kg) divided by height (m^2^) squared. Overweight was defined as BMI ≥25 kg/m^2^ and obesity as BMI ≥30 kg/m^2^. Additionally, we assessed waist circumference (WC), body fat percentage (BFP) and visceral fat area (VFA) in NFBC1966. Waist circumference (cm) was measured from the point midway between the costal margin and iliac crest. Body fat (%) and visceral fat area (cm^2^) were measured by bio-impendence using InBody 720.

### Covariates

Known early life risk factors for obesity were used as covariates. In HBCS1934–1944, maternal weight (kg) and height (cm) data were taken from hospital records at the time when women came to the hospital to deliver. In NFBCs, maternal weight and height before pregnancy were derived from questionnaires and maternal BMI was calculated as described earlier.

We calculated a polygenic risk score for BMI (PRS BMI) for each HBCS1934–1944, NFBC1966 and NFBC1986 individual as a weighted sum of BMI-increasing alleles at genome-wide single nucleotide polymorphisms (SNPs). Genotype quality control for NFBC1966 is presented in Additional file 1, text 1. For SNP weights we used the beta coefficients estimated by the BOLT-LMM model using the “--predBetasFile” flag in the BOLT-LMM software package [20]. We estimated BOLT-LMM SNP effects in the UK Biobank (UKB), a prospective cohort of 502,628 volunteers recruited across the UK at age 40–69 years through United Kingdom National Health Service registers [21, 22]. To calculate PRS we used PRSice version 2.1.3.beta [23], which automatically harmonises the base (UKB) and target (HBCS1934–1944, NFBC1966, NFBC1986) data sets and removes ambiguous (A/T and C/G) SNPs, and calls the plink --score function [24]. More detailed information in Additional file 1, text 1.

### Statistical analysis

Information from study participants was used to create the composite social disadvantage score and assign the exposure of individuals to high, intermediate or low social disadvantage. We selected our core study sample for each cohort based on those who had an early life social disadvantage score and measured BMI at the relevant age. Descriptive statistics were generated for explanatory and outcome measures and distributions were examined for normality. Skewed variables were logarithmically transformed. Univariable linear regression was used to assess the association of each explanatory variable with the primary outcome i.e. BMI at follow-ups.

In HBCS1934–1944 all analyses were conducted using Stata/MP 15.5 (StataCorp, 4905 Lakeway Dr., College Station, TX 77845, USA). In NFBCs factor analysis was conducted using Mplus 7.0 [25] and other analysis using SAS Enterprise 7.15 (2017, SAS Institute Inc. Cary, NC, USA). Mplus uses full information maximum likelihood method to estimate the model parameters in order to account for missing data [26]. In Stata, complete case analysis was used to create the factor scores within the HBCS1934–1944. Factor scores were extracted and used in univariable and multivariable linear regression models to assess associations between social disadvantage and BMI and other measures of body composition. These models were adjusted for PRS BMI and ancestry principal components, maternal BMI and sex. In regression analysis, individuals without data for PRS BMI or maternal BMI were excluded so numbers were the same for each model allowing comparison.

## Results

### Population characteristics

In HBCS1934–1944, at a mean age of 62 years, mean BMI was 27.7 kg/m^2^(SD 4.8) and prevalence of overweight and obesity were 44.4 and 26.8%, respectively. At 46-years in the NFBC1966, mean BMI was 26.9 kg/m^2^(SD 4.9) and 39.4 and 21.4% were overweight and obese, respectively. In NFBC1986 at the age of 16-years, mean BMI was 21.2 kg/m^2^(SD 3.5) (Table 1) and 8.9 and 2.7% were overweight and obese, respectively. Additionally, there were no difference in early life exposure to social disadvantage in those who attended the later follow-up and those who did not in both NFBC1966 (*p* = 0.19) and NFBC1986 (*p* = 0.36) [results not shown in main tables]. The retrospective design of the HBCS1934–1944 does not allow a measure of non-participation according to early exposure. As shown in Fig. 1, the absence of measures of early social disadvantage created attrition in the final sample.

**Table 1:** Descriptives for HBCS1934–1944, NFBC1966 and NFBC1986

	HBCS1934–1944 (*n* = 766–1277)	NFBC1966 (*n* = 5803–5807)	NFBC1986 (*n* = 6717)
Sex			
Men	577 (45)	2553 (44)	3317 (49)
Women	700 (55)	3254 (56)	3400 (51)
BMI 16 (kg/m^2^)	–	–	21.19 (3.50)
BMI 46 (kg/m^2^)	–	26.86 (4.90)	–
BMI 62 (kg/m^2^)	27.7 (4.81)	–	–
Maternal BMI (kg/m^2^)	26.5 (2.9)	23.16 (3.18)	22.33 (3.38)
PRS BMI^a^	0.003 (1.00)	8.62^−15^ (1.00)	0.006 (1.00)
Change in Social Disadvantage			
Stable SD	322 (42)	2535 (44)	–
Reduced SD	196 (26)	1426 (25)	–
Increased SD	248 (32)	1842 (32)	–

^a^PRSBMI = in polygenic risk score for body mass index standardized values used

**Fig. 3:**
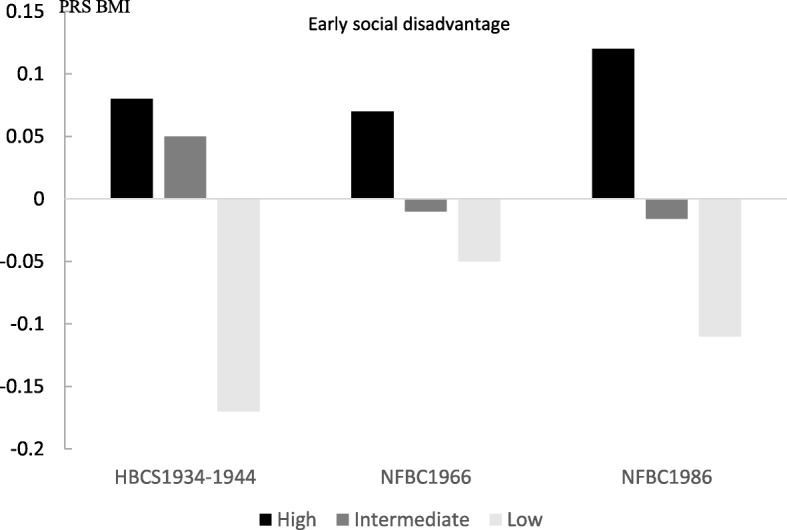
Polygenic risk score for BMI (PRS BMI) by early social disadvantage tertile for each cohort

In HBCS1934–1944, individuals exposed to high early social disadvantage also had the highest mean BMI in later life (Table 2). In the more recent NFBCs, later life BMI was related to early life social disadvantage in a gradual manner with the highest social disadvantage group having the highest BMI. Maternal BMI was also highest in the group exposed to high early social disadvantage in both NFBCs. Importantly, we observed a difference in the PRS BMI scores in all cohorts, indicating that individuals exposed to high early social disadvantage had increased genetic predisposition for greater BMI (Fig. 3). In NFBC1966, high early social disadvantage was also associated with high later life VFA (Additional file 1, Table 3). In women, a similar relationship was observed for WC and BFP, but not in men (Additional file 1, Table 3).

**Table 2:** Descriptives by early social disadvantage in Helsinki Birth Cohort 1934–1944 (HBCS1934–1944), Northern Finland Birth Cohort 1966 (NFBC1966) and Northern Finland Birth Cohort 1986 (NFBC1986)

HBCS1934–1944 Early social disadvantage										NFBC1966 Early social disadvantage													NFBC1986 Early social disadvantage							
	High			Intermediate			Low				High			Intermediate			Low					High		Intermediate			Low			
	n	%		n		%	n	%			n	%		n		%	n		%			n	%	n	%		n	%		
	398	31		561		44	318	25			1463	25		2900		50	1444		25			1682	25	3363	50		1672	25		
	n	$\overline{x}$	sd	n	$\overline{x}$	sd	n	$\overline{x}$	sd	p.	n	$\overline{x}$	sd	n	$\overline{x}$	sd	n	$\overline{x}$	sd	p.	n	$\overline{x}$	sd	n	$\overline{x}$	sd	n	$\overline{x}$	sd	p.
BMI	398	28.2	5.5	561	27.4	4.5	318	27.7	4.3	0.046	1463	27.3	5.0	2900	26.8	4.9	1444	26.5	4.7	< 0.001	818	21.5	3.8	1625	21.4	3.7	779	21.0	3.1	0.014
Mat BMI	366	26.6	3.0	508	26.5	2.8	273	26.5	3.2	0.67	1276	23.7	3.5	2667	23.2	3.2	1398	22.6	2.7	< 0.001	818	22.5	3.7	1625	22.3	3.5	779	21.9	2.7	< 0.001
Sex	n	%		n		%	n	%		0.018	n	%		n		%	n		%	0.019		n	%	n	%		n	%		0.612
Men	176	31		236		41	165	29			619	42		1254		43	680		47			408	49.9	776	47.8		378	48.5		
Women	222	31		325		46	153	22			844	58		1646		57	764		53			410	50.1	849	52.3		401	51.5		

^a^BMI was measured at age of approximately 62 years in HBCS1934–1944, 46 in NFBC1966 and 16 in the NFBC1986

^b^Mat BMI = maternal body mass index measured during late pregnancy in HBCS1934–1944

^c^PRS BMI = in polygenic risk score for body mass index standardized values used

**Fig. 4:**
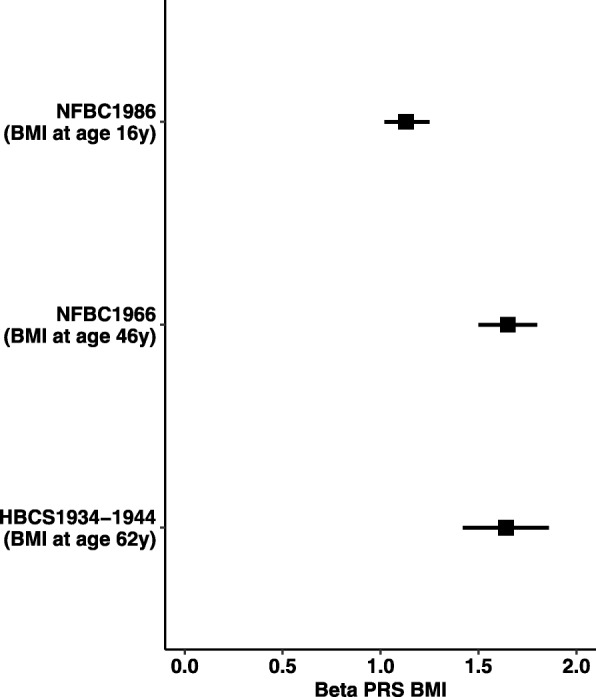
Association of polygenic risk scores for BMI (PRS BMI) with BMI approximately at 62 years in Helsinki Birth Cohort Study 1934–1944 (HBCS1934–1944), at 46 years in Northern Finland Birth Cohort 1966 (NFBC1966) and at 16 years in Northern Finland Birth Cohort 1986 (NFBC1986). *In PRS BMI standardized values are used; β can be interpreted as SD change in BMI per 1-SD increase in PRS BMI*

The PRS BMI explained10–12% of the total variation in later life BMI when adjusted for sex in all cohorts (Fig. 4). Due to differences in PRS BMI between social disadvantage groups, we also controlled for population stratification by adjusting the top five principal components in NFBC1966 [27] and four multidimensional-scaling coordinates in NFBC1986.

### Association of early life social disadvantage with later life body mass index and other measures of adiposity

**Fig. 5:**
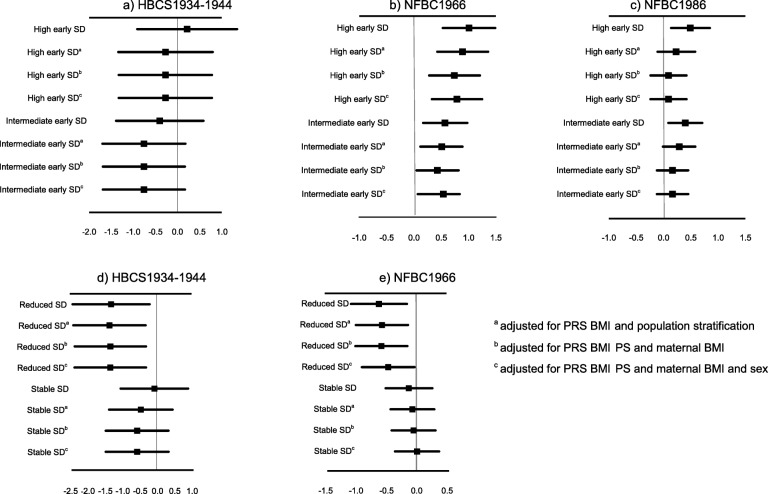
Early social disadvantage and its association with later life BMI (β, 95% CIs) **a** in Helsinki Birth Cohort Study (HBCS1934–1944, *n* = 533), **b** in Northern Finland Birth Cohort 1966 (NFBC1966, *n* = 3354) and **c** in Northern Finland Birth Cohort 1986 (NFBC1986, *n* = 3222, low social disadvantage group was set as a reference) and social mobility during lifecourse and BMI **d** in HBCS1934–1944 (n = 533) and **e** in NFBC1966, (*n* = 3353, increased social disadvantage was set as a reference)

In HBCS1934–1944, despite observing a trend for higher adult BMI in those exposed to high early life social disadvantage, these individuals were not found to be at greater risk of increased BMI (β 0.22, 95% CI –0.91,1.35) compared to those with low early social disadvantage (Fig. 5). However, exposure to high (β 0.79, 95% CI 0.33, 1.25) or intermediate early life social disadvantage (β 0.54, 95% CI 0.07, 0.84) was related to higher later life BMI even after full adjustments in NFBC1966 (Fig. 5). In the youngest cohort, NFBC1986, the association between high (β 0.29, 95% CI –0.01,0.58) and intermediate early social disadvantage(β 0.23, 95% CI –0.11,0.58) with higher later life BMI was attenuated with the addition of PRS BMI (Fig. 5).

In addition, results concerning early exposure to social disadvantage and later life VFA were similar to later life BMI in NFBC1966 (Additional file 1, Table 4). Sex-specific analyses for later life WC and BFP showed contrasting results. Women exposed to high or intermediate early social disadvantage were at greater risk of higher WC and BFP in adulthood after adjustments. Conversely, in men early social disadvantage was not associated with adult WC or BFP (Additional file 1, Table 5).

### Social mobility and its association with later life body mass index and other measures of adiposity

In HBCS1934–1944, we observed that 42.0% of individuals (*n* = 322) remained stable in their lifecourse exposure to social disadvantage,25.6% (*n* = 196) were upwardly mobile and the remaining 32.4% (*n* = 248) were downwardly mobile. In NFBC1966, corresponding figures were 43.7% (*n* = 2535), 24.6% (*n* = 1426) and 31.7% (*n* = 1842), respectively. In HBCS1934–1944, there was no difference in social mobility between sexes. In NFBC1966, a greater proportion of women were found to remain stable (25.2%) or show upward mobility (16.6%) compared to men (18.5, 8.0%, *p* < 0.001, respectively). There was no difference in maternal BMI or PRS BMI according to social mobility in either cohort.

In both HBCS1934–1944 and NFBC1966, participants showing upward social mobility over the lifecourse had lower later life BMI after adjustment for all covariates (Fig. 5). Additional file 1, Tables 6-7 show results on other measures of adiposity in NFBC1966. Women showing upward social mobility during the lifecourse also had lower later life WC and BFP after adjustments (Additional file 1, Table 7).

## Discussion

In this longitudinal study of three generations of birth cohorts, we found that participants exposed to high and intermediate social disadvantages in early life had higher BMI in later life than those who had been born into families with low social disadvantage. In NFBC1966, this association persisted following adjustment for PRS BMI (as well as principal components, in order to control for population stratification), maternal BMI and sex. However, in NFBC1986 the association was attenuated by the adjustment for PRS BMI. We observed a similar trend in HBCS1934–1944 for higher later life BMI in the group exposed to high early social disadvantage. Furthermore, individuals showing upward social mobility during the lifecourse had lower later life BMI compared to individuals with downward social mobility based on findings in our two adult cohorts (HBCS1934–1944 and NFBC1966).

These three birth cohorts reflect changes in the socio-historical context which may explain the trends we are observing. When individuals in the oldest birth cohort were born (1934–1944), Finland was an agricultural society. At this time, food was scarce as they moved from the Great Depression into a period of war when groceries were rationed. During the 1960’s, Finland was transitioning into an industrial economy, characterised by urbanization and mass migration. When the youngest birth cohort was born in the 1980’s, Finland was living in an economic boom and becoming a consumer nation [28].

A previous study on seven population-based surveys from six countries showed that in cohorts born between 1910 and 1961, women from manual childhood backgrounds had an elevated risk of obesity in adulthood, although it was attenuated in two studies after adjustment for adult socioeconomic position. Among men, the association with childhood position was mostly in the same direction, although effects were weaker [29], which is consistent with what we observed in HBCS1934–1944.

NFBC1966 showed the most robust association between early social disadvantage and later life BMI, which is also consistent with previous reports showing the importance of occupational social class of the head of household in early life and risk for adult obesity [30] even after adjustment for parental BMI in men [9]. Additionally, our results on early social disadvantage and adult VFA (Additional file 1, Table 4) and adult WC and BFP in women showed similar trends to that of BMI (Additional file 1, Table 5).

In our youngest cohort included in the study (NFBC1986) the association between early social disadvantage and later life BMI was attenuated after adjusting for genetic predisposition to higher BMI. Interestingly, we also found that in all cohorts the PRS BMI was not equally distributed at birth and was highest in individuals born into the group with highest early life social disadvantages. This may be partly explained by social selection [31], meaning that parents’ of the participants with higher genetic predisposition to higher BMI may have already drifted to lower social positions and have difficulties in rising to higher social positions. There is also evidence that individuals tend to seek the company of people, who have similar characteristics than their own e.g. for example marrying people of the same level of educational attainment [32]. These two observed trends of unequal distribution of PRS BMI at birth and stronger effect of genetic risk through time strongly suggest that the current obesogenic environments may be promoting the vicious cycle between low social class and poor health outcomes. We found one other similar study which has demonstrated interaction between genetic influence and socio-economic status in change in BMI across adolescence [12]. Finally, it could be hypothesised that BMI may exert a causal influence on psychosocial factors which may in turn contributed to the observed association. This may be consistent with the finding that obesity may be causally associated to smoking [33] and future research based on multivariate mendelian randomisation with very large sample size, for example, may help elucidating the observed associations.

Individuals born in the two oldest cohorts with reduced exposure to social disadvantage during their lifecourse, also had lower BMI compared to individuals with increased exposure to social disadvantage. This is in line with previous studies in women which have also shown that upward mobility from low social origins to higher position during the lifecourse have decreased the risk for obesity compared to those with a stable situation [34, 35]. Therefore, a society’s degree of social mobility is important, for example in immobile societies an individual’s education is strongly related to their parent’s education [36].

It has not been possible to test the pathways of effect between high early social disadvantage and later life BMI in the present study. However, we would speculate that underlying factors such as stress [37], nutrition in infancy and childhood, psychological factors such as emotional deprivation and social norms regarding dietary factors and obesity [10] may be involved. Furthermore, it has been shown that childhood social disadvantage was related to smoking and excess alcohol intake in later life [38] and therefore could foster other unhealthy lifestyle behaviors leading to obesity.

Strengths of our study include the use of longitudinal or register-based measures of social disadvantage in early life and adulthood to avoid recall bias and construct comprehensive measures of social disadvantage reflecting different social variables [8]. Previously, early social disadvantage has been solely based on a single variable, generally father’s occupation. We have already demonstrated with the initial step in our variable selection strategy that there are a number of variables associated with later life BMI and potentially acting via different pathways of effect. Therefore, we wanted to capture the complexities of social construct by composing a composite variable. Use of this tested systematic strategy allows comparison between different datasets and time points as the selection process is carried out for each composite measure and thus accounts for differences in variable importance over time and by region. This study also examines the relationship between early social disadvantage and later life obesity in three different birth cohorts, which is especially valuable given the trends of intergenerational transmission of obesity and impact of maternal obesity [39, 40]. Additionally, we were able to use objective measures of body composition, including VFA, WC and BFP in the NFBC1966 in addition to BMI, which has been criticized for not differentiating between body lean mass and body fat mass [41].

We do acknowledge that our study has some limitations. Although the use of three separate cohorts allows us the opportunity to study trends and replicate findings, there are also challenges with harmonisation. HBCS1934–1944 used a retrospective identification of participants based on birth records and subsequent follow-up was primarily via register-based data. Due to availability of funding, a small random sample was invited to attend a clinical examination in later life which resulted in a considerably smaller number of participants included in the present study, particularly in analysis of social mobility. Participants of the cohorts were of different ages at the time of the measurement of BMI and maternal BMI was measured during late pregnancy in the HBCS1934–1944 and not in pre-pregnancy as in NFBCs. We did not conduct meta-analysis due to these differences and high expected heterogeneity of the studies [42]. Whilst the PRS for BMI explained only about 10–12% of variation of BMI, this is expected with the methodology used and similar to the variation explained in similar studies [43]. In addition, we have used ranking of participants within their study populations in order to compare how their position within society has changed over time, however we have not been able to account for changes in environment during this period, which is an inherent limitation of longitudinal studies exploring social factors. Social mobility was not available in NFBC1986 as adult data is not yet available for this cohort.

We conducted sensitivity analyses for NFBC1966 data using only the early social disadvantage measures used in the HBCS1934–1944 (instead of number of persons in room we used material wealth: home ownership, car, electricity, telephone, running water and television). After adjustment for PRS BMI the association between high early social disadvantage and BMI was still significant, but intermediate exposure was no longer associated with later life BMI. It may be that the effect of early social disadvantage on later BMI is better captured with a range of variables. We used a systematic approach for variables selection and reduction according to the association of the social variables and later BMI. This approach allowed us to reduce the number of variables included in the factor score, which can also improve the interpretability as well as replicability across cohorts. We must acknowledge that this process may remove some variables that may contribute to latent factors and further research, probably based in only one cohort should explore other methods to include more social factors that may influence the risk of obesity.

## Conclusions

Our results based on three generations of birth cohorts showed that participants exposed to high social disadvantage in early life had a tendency towards higher BMI in later life. This association was most robust in the cohort born in the 1960’s, withstanding adjustment for genetic predisposition and maternal BMI. The genetic effect was greatest on those born into a more obesogenic environment in the 1980s. Notably, our results based on the two adult cohorts showed that participants with reduced exposure to social disadvantage during the lifecourse had a lower BMI than those with increased exposure, suggesting a potential pathway for intervention.

## Supplementary information

**Additional file 1: Table 1A.** Variable selection in Helsinki Birth Cohort Study 1934–1944 (✓ included, ✗ excluded). **Table 1B**. Variable selection in Northern Finland Birth Cohort 1966 (✓ included, ✗ excluded). **Table 1C**. Variable selection in Northern Finland Birth Cohort 1986 (✓ included, ✗ excluded). **Table 2**. Social disadvantage variables with their original categorizations and coding used within confirmatory factor analysis in early life in Helsinki Birth Cohort Study 1934–1944 (HBCS1934–1944), Northern Finland Birth Cohort 1966 (NFBC1966), and Northern Finland Birth Cohort Study 1986 (NFBC1986) and at age of 44-years in HBCS1934–1944 and 46-years in NFBC1966. **Text 1**. Genotype quality control for NFBC1966 and more detailed information concerning calculation of polygenic risk score for body mass index (BMI). **Table 3**. Association of early social disadvantage with visceral fat area (cm^2^), waist circumference (cm) and body fat (%) at 46-years in Northern Finland Birth Cohort 1966 (NFBC1966). **Table 4.** Association between early social disadvantage with visceral fat area (cm^2^) at age of 46-years in Northern Finland Birth Cohort 1966 (NFBC1966, *n* = 3294). Low social disadvantage was set as a reference group. **Table 5**. Associations between early social disadvantage with waist circumference (cm) and body fat (%) at age of 46-years in men and in women in Northern Finland Birth Cohort 1966 (NFBC1966). Low social disadvantage was set as a reference group. **Table 6**. Association of change in social disadvantage during the life-course with visceral fat area (cm^2^) at age of 46-years in Northern Finland Birth Cohort (NFBC1966, *n* = 3293). Increased social disadvantage was set as a reference group. **Table 7**. Association of change in social disadvantage during the lifecourse with waist circumference (cm) and body fat (%) at age of 46-years in men and women in Northern Finland Birth Cohort 1966 (NFBC1966). Increased social disadvantage was set as a reference group. **Figure 1.** Directed acyclic diagram (DAG) for the tested association. We may hypothesise the association between exposure to early social disadvantage to be the result of co-existing pathways. This includes the possible interplay with the child polygenic risk score for BMI that might in part proxy some confounding effects of his/her parents’ BMI.

## Data Availability

The data that support the findings of this study are available, but restrictions apply to the availability of these data, which were used under license for the current study, and so are not publicly available. Data are however available upon reasonable request and with permission.

## Abbreviations

BFP: Body fat percentage
CFA: Confirmatory factor analysis
HBCS1934–1944: Helsinki Birth Cohort Study 1934–1944
ID: Personal identity number
NFBC: Northern Finland Birth Cohort
PC: Principal component
PRS BMI: Polygenic risk score for body mass index
SNPs: Single nucleotide polymorphisms
UKB: UK Biobank
VFA: Visceral fat area
WC: Waist circumference
