# Supplementary material for: Early exposure to social disadvantages and later life body mass index beyond genetic predisposition in three generations of Finnish birth cohorts
Source: BMC Public Health. 2020 May 18;20:708. doi: 10.1186/s12889-020-08763-w (PMC7236362; doi:10.1186/s12889-020-08763-w)

Additional file 1

Table 1A: Variable selection in Helsinki Birth Cohort Study1934-1944 (✓ included, ✗ excluded).

| Explanatory variables at early life n=2001 | | | | |
| --- | --- | --- | --- | --- |
|  | Descriptives | Association with BMI at 62 years | |  |
| Variables | N (%) | Estimate (Beta, 95% CI) | p-value | Inclusion |
| Father'shighestachieved SES |  |  |  | **✓** |
| Professionals | 342 (17) | Ref. |  |  |
| Junior clericals | 453 (23) | 0.66 (0.003, 1.32) | 0.049 |  |
| Workers | 1192 (60) | 1.07 (0.51, 1.63) | 0.000 |  |
| Mother'smarital status |  |  |  | **✗** |
| Married | 1938 (97) | Ref. |  |  |
| Unmarried | 55 (3) | -1.03 (-2.29, 0.22) | 0.107 |  |
| Other | 8 (0.4) | -0.7 (-3.95, 2.56) | 0.674 |  |
| Mother'shighestachieved SES |  |  |  | **✓** |
| Professionals | 81 (4) | Ref. |  |  |
| Junior clerical | 641 (32) | 0.45 (-0.63, 1.53) | 0.417 |  |
| Workers | 1279 (64) | 0.99 (-0.06, 2.04) | 0.066 |  |
| Number of rooms in a household |  |  |  | **✗** |
| 1 room | 623 (43) | Ref. |  |  |
| 2 rooms | 606 (41) | -0.22 (-0.76, 0.32) | 0.432 |  |
| 3 rooms | 182 (12) | -0.47 (-1.26, 0.33) | 0.249 |  |
| 4 rooms | 39 (3) | -1.83 (-3.39, -0.27) | 0.022 |  |
| 5 rooms | 9 (1) | 0.96 (2.21, 4.14) | 0.552 |  |
| 6 rooms | 1 (0.1) | -2.86 (-12.32, 6.6) | 0.553 |  |
| 8 rooms | 1 (0.1) | -4.45 (-13.91, 5.01) | 0.357 |  |
| Size of household-dwelling units (number of persons) |  |  |  | **✗** |
| 2 persons | 24 (2) | Ref. |  |  |
| 3 persons | 570 (44) | 0.66 (-1.3, 2.62) | 0.507 |  |
| 4 persons | 415 (32) | 0.83 (-1.15, 2.8) | 0.412 |  |
| 5 persons | 185 (14) | 0.37 (-1.67, 2.41) | 0.721 |  |
| 6 persons | 66 (5) | 1.83 (-0.41, 4.08) | 0.109 |  |
| 7 persons | 19 (1) | 1.25 (-1.65, 4.14) | 0.398 |  |
| 8 persons | 16 (1) | 0.34 (-2.7, 3.37) | 0.829 |  |
| 9 persons | 3 (0.2) | -0.67 (-6.43, 5.09) | 0.820 |  |
| 10 persons | 2 (0.2) | 1.17 (-5.75, 8.1) | 0.740 |  |
| People per room | 1288 | 0.29 (0.06, 0.53) | 0.013 | **✓** |
|  | | | | |
| Explanatory variables at 44 years n=2001 | | | | |
| Marital status |  |  |  | **✗** |
| Married | 1555 (78) | Ref. |  |  |
| Other | 435 (22) | 0.18 (-0.32, 0.68) | 0.474 |  |
| Highereducation |  |  |  | **✓** |
| University | 387 (31) | Ref. |  |  |
| Vocational | 28 (2) | 0.62 (-1.14, 2.38) | 0.490 |  |
| No highereducation | 843 (67) | 1.17 (0.62, 1.72) | 0.000 |  |
| Householdincome |  |  |  | **✓** |
| Rank 1 (highest) | 508 (26) | Ref. |  |  |
| Rank 2 | 461 (23) | 0.55 (-0.04, 1.14) | 0.067 |  |
| Rank 3 | 399 (20) | 1.1 (0.48, 1.71) | 0.000 |  |
| Rank 4 | 357 (18) | 0.83 (0.19, 1.46) | 0.011 |  |
| Rank 5 (lowest) | 259 (13) | 0.95 (0.25, 1.65) | 0.008 |  |
| Highestachieved SES |  |  |  | **✓** |
| Highofficial | 618 (32) | Ref. |  |  |
| Lowofficial | 876 (46) | 0.36 (-0.12, 0.83) | 0.146 |  |
| Self-employed | 115 (6) | 1.33 (0.41, 2.26) | 0.005 |  |
| Labourers | 308 (16) | 1.4 (0.77, 2.04) | 0.000 |  |
| Size of household-dwelling units (number of persons) |  |  |  | **✗** |
| 0 persons | 6 (0.3) | Ref. |  |  |
| 1 person | 149 (7) | -2.83 (-6.65, 0.99) | 0.146 |  |
| 2 persons | 383 (19) | -2.66 (-6.43, 1.12) | 0.168 |  |
| 3 persons | 515 (26) | -3.25 (-7.01, 0.52) | 0.091 |  |
| 4 persons | 678 (34) | -3.37 (-7.13, 0.39) | 0.079 |  |
| 5 persons | 208 (10) | -2.56 (-6.36, 1.24) | 0.187 |  |
| 6 persons | 33 (2) | -3.18 (-7.25, 0.89) | 0.126 |  |
| 7 persons | 8 (0.4) | -1.08 (-6.03, 3.87) | 0.669 |  |
| 8 persons | 6 (0.3) | -1.02 (-6.31, 4.28) | 0.707 |  |
| 9 persons | 4 (0.2) | 1.76 (-4.16, 7.68) | 0.560 |  |
| Number of rooms in a household |  |  |  | **✗** |
| 1 room | 99 (5) | Ref. |  |  |
| 2 rooms | 326 (17) | 0.27 (-0.78, 1.33) | 0.611 |  |
| 3 rooms | 540 (28) | -0.18 (-1.18, 0.83) | 0.732 |  |
| 4 rooms | 572 (29) | -0.27 (-1.27, 0.73) | 0.596 |  |
| 5 rooms | 287 (15) | -0.67 (-1.74, 0.41) | 0.222 |  |
| 6 rooms | 86 (4) | -0.59 (-1.94, 0.77) | 0.398 |  |
| 7 rooms | 26 (1) | -0.5 (-2.53, 1.53) | 0.629 |  |
| 8 rooms | 4 (0.2) | 1.3 (-3.4, 6) | 0.588 |  |
| 9 rooms | 3 (0.2) | 2.94 (-2.45, 8.34) | 0.285 |  |
| People per room | 1943 | 0.48 (-0.002, 0.95) | 0.051 | **✗** |

All regression analyses are unadjusted.

Table 1B: Variable selection in Northern Finland Birth Cohort 1966 (✓ included, ✗ excluded).

| Explanatory variables at early life n=5828 | | | | | |
| --- | --- | --- | --- | --- | --- |
| Variables | Descriptives | | Association with BMI at 46 years | | Inclusion |
|  | N (%) | | Estimate (Beta, 95% CI) | p-value |  |
| Parentalmarital status |  | |  |  | **✓** |
| Married | 5611 (97) | | Ref. |  |  |
| Unmarried/divorced/widow | 188 (3) | | 1.01 (0.30, 1.72) | 0.006 |  |
| Maternaloccupation |  | |  |  | **✓** |
| Professional | 773 (14) | | Ref. |  |  |
| Skilledworkers | 1265 (22) | | 0.38 (-0.05, 0.82) | 0.09 |  |
| Farmer/farmer’swife | 3240 (57) | | 0.54 (0.16, 0.92) | 0.006 |  |
| Unskilledworkers | 445 (8) | | 0.91 (0.34,1.48) | 0.002 |  |
| Paternaloccupation |  | |  |  | **✓** |
| Professional | 1455 (26) | | Ref. |  |  |
| Skilledworkers | 1846 (33) | | 0.44 (0.11, 0.78) | 0.01 |  |
| Farmer | 1174 (21) | | 0.31 (-0.06, 0.69) | 0.10 |  |
| Unskilledworkers | 1077 (19) | | 0.68 (0.30, 1.07) | 0.001 |  |
| No occupation | 36 (1) | | 0.64 (-0.97,2.26) | 0.43 |  |
| Materialwealth |  | |  |  | **✓** |
| Rank 0 (highest) | 215 (4) | | Ref. |  |  |
| Rank 1 | 632 (12) | | 0.07 (-0.69, 0.83) | 0.85 |  |
| Rank 2 | 1065 (21) | | 0.28 (-0.44, 0.99) | 0.45 |  |
| Rank 3 | 1275 (25) | | 0.56 (-0.15, 1.27) | 0.12 |  |
| Rank 4 | 1145 (22) | | 0.71 (-0.01, 1.42) | 0.05 |  |
| Rank 5 | 701 (14) | | 0.80 (0.05, 1.55) | 0.04 |  |
| Rank 6 (lowest) | 149 (3) | | 1.24 (0.22, 2.27) | 0.02 |  |
| Maternaleducation |  | |  |  | **✓** |
| Matriculation+ | 296 (5) | | Ref. |  |  |
| Vocational | 1758 (31) | | 0.30 (-0.30, 0.90) | 0.33 |  |
| Primaryonly | 3667 (64) | | 0.83 (0.25, 1.41) | 0.01 |  |
|  |  | |  |  |  |
| Explanatory variables at 46 years n=5828 | | | | | |
| Marital status |  |  | |  | **✗** |
| Married/civil part/cohabiting | 4354 (79) | Ref. | |  |  |
| Single/divorced/widow | 1171 (21) | 0.20 (-0.11, 0.52) | | 0.20 |  |
| Basic education |  |  | |  | **✓** |
| Matriculation | 2522 (46) | Ref. | |  |  |
| No matriculation | 2989 (54) | 1.20 (0.94, 1.45) | | <0.001 |  |
| Highereducation |  |  | |  | **✓** |
| University | 1533 (29) | Ref. | |  |  |
| Vocational | 3655 (68) | 1.10 (0.82, 1.40) | | <0.001 |  |
| No highereducation | 183 (3) | 2.27 (1.53, 3.01) | | <0.001 |  |
| Occupation |  |  | |  | **✓** |
| Professional | 3799 (67) | Ref. | |  |  |
| Manualworker/farmer | 1112 (20) | 0.94 (0.61, 1.26) | | <0.001 |  |
| Notcurrentlyworking | 768 (14) | 1.01 (0.63, 1.38) | | <0.001 |  |
| Employmenthistory |  |  | |  | **✗** |
| Mostlyemployed | 5300 (97) | Ref. | |  |  |
| Mostlyunemployed | 181 (3) | 0.33 (-0.39, 1.05) | | 0.37 |  |
| Employment status |  |  | |  | **✓** |
| Employed | 4840 (88) | Ref. | |  |  |
| Not in labour force | 389 (7) | 1.11 (0.61, 1.61) | | <0.001 |  |
| Unemployed | 267 (5) | 0.51 (-0.09, 1.11) | | 0.10 |  |
| Home ownership |  |  | |  | **✓** |
| Home ownership | 4660 (85) | Ref. | |  |  |
| No home ownership | 847 (15) | 0.82 (0.47, 1.18) | | <0.001 |  |
| Householdincome |  |  | |  | **✗** |
| Rank 1 (highest) | 584 (20) | Ref. | |  |  |
| Rank 2 | 571 (20) | 0.33 (-0.19, 0.86) | | 0.22 |  |
| Rank 3 | 595 (21) | 0.69 (0.17, 1.21) | | 0.01 |  |
| Rank 4 | 558 (19) | -0.06 (-0.59, 0.47) | | 0.82 |  |
| Rank 5 (lowest) | 575 (20) | -0.28 (-0.80, 0.25) | | 0.30 |  |

All regression analyses are unadjusted.

Table 1C: Variable selection in Northern Finland Birth Cohort 1986 (✓ included, ✗ excluded).

| Explanatory variables at early life n = 6764 | | | | |
| --- | --- | --- | --- | --- |
| Variables | Descriptives | Association with BMI at 16 years | | Inclusion |
|  | N (%) | Estimate (Beta, 95% CI) | p-value |  |
| Parentalmarital status |  |  |  | **✗** |
| Married/co-habiting | 6444 (95) | Ref. |  |  |
| Single/divorced/widow | 306 (5) | 0.31 (-0.10, 0.71) | 0.13 |  |
| Maternaloccupation |  |  |  | **✓** |
| Professional | 1835 (28) | Ref. |  |  |
| Skilledworkers | 2639 (40) | 0.29 (0.08, 0.49) | 0.007 |  |
| Farmer/farmer’swife | 334 (5) | 1.27 (0.86,1.68) | <0.001 |  |
| Unskilledworkers | 1757 (27) | 0.19 (-0.04,0.42) | 0.11 |  |
| Paternaloccupation |  |  |  | **✓** |
| Professional | 2219 (35) | Ref. |  |  |
| Skilledworkers | 3069 (48) | 0.31 (0.12, 0.50) | 0.001 |  |
| Farmer | 453 (7) | 0.69 (0.34, 1.04) | 0.001 |  |
| Unskilledworkers | 664 (10) | 0.16 (-0.14,0.46) | 0.31 |  |
| Materialwealth |  |  |  | **✓** |
| Rank 0 (highest) | 437 (7) | Ref. |  |  |
| Rank 1 | 2256 (37) | 0.02 (-0.34, 0.38) | 0.93 |  |
| Rank 2 | 1652 (27) | -0.13(-0.50, 0.24) | 0.50 |  |
| Rank 3 | 913 (15) | -0.04 (-0.44,0.36) | 0.85 |  |
| Rank 4 | 469 (8) | 0.24 (-0.22,0.70) | 0.30 |  |
| Rank 5 | 172 (3) | 0.13 (-0.49, 0.75) | 0.67 |  |
| Rank 6 | 83 (1) | 0.48(-0.34, 1.31) | 0.25 |  |
| Rank 7 | 85 (1) | 0.94 (0.12,1.75) | 0.02 |  |
| Rank 8 (lowest) | 52 (1) | 0.09 (-0.91,1.10) | 0.85 |  |
| Maternaleducation |  |  |  | **✓** |
| Matriculation + | 1814 (31) | Ref. |  |  |
| Vocational | 2688 (45) | 0.37 (0.16,0.58) | 0.005 |  |
| Primaryonly | 1439 (24) | 0.48 (0.24,0.73) | 0.001 |  |

All regression analyses are unadjusted.

Table 2. Social disadvantage variables with their original categorizations and coding used within confirmatory factor analysis in early life in Helsinki Birth Cohort Study1934-1944 (HBCS 1934-1944), Northern Finland Birth Cohort 1966 (NFBC1966), and Northern Finland Birth Cohort Study 1986 (NFBC1986) and at age of 44-years in HBCS1934-1944 and 46-years in NFBC1966.

| HBCS |  | NFBC 1966 | | NFBC 1986 | |
| --- | --- | --- | --- | --- | --- |
| Original categorizations | Categorizations used | Original categorizations | Categorizations used | Original categorizations | Categorizations used |
| Early life | | Early life | | Early life | |
| - | - | Parental marital status   1. married 2. unmarried 3. widowed 4. divorced | 1. married  2. unmarried, divorced, widowed | - | - |
| - | - | Maternal education  1.none or circulating school  2.1-4 years of elementary school  3. 5-8 years of elementary school or part of the secondary school  4. ½ -2 years of vocational school  5. more than 2 years of vocational school  6. secondary school  7 secondary school and more  8. matriculation examination  9. matriculation examination and more | 1. matriculation examination or more  2. vocational school or secondary school and more (at least ½ year of vocational school)  3. primary only (none or 1-8 years of elementary school or part of the secondary school) | Maternal education  1. <6 of primary school  2. 7-8 years primary school  3. 9-10 years primary school  4. vocational school or college 6-12 months  5. vocational school > 1year or college  6. matriculation, no vocational schooling  7. matriculation +college  8. matriculation, university studies not finished  9. university degree | 1. matriculation+  2. vocational  3. primary only |
| Maternal occupation   1. Housewives 2. Employers 3. Self-employed 4. Senior clericals 5. Junior clericals 6. Workers 7. Pensioners 8. Students   8. Others | 1. Professionals/Senior clericals – Employers, Self-employed, Senior clericals  2. Junior clericals  3. Workers | Nine response categories were conducted based on the question concerning maternal occupation:   1. no occupation (housewife) 2. upper white collar 3. lower white collar 4. skilled worker 5. unskilled worker 6. farmer, field area ≥8 ha 7. farmer, field area <8 ha 8. farmers’s wife, field area ≥8ha 9. farmers’ wife, field area <8 ha | 1. professional – upper and lower white-collar  2. skilled worker  3. farmer/farmer’s wife/housewife  4. unskilled worker | Ten response categories concerning maternal occupation:  1. unskilled or apprentice  2. skilled manual  3.skilled non-manual  4. professional  5. entrepreneur  6. farmer  7. student  8.at home  9. sick pension  10. unemployed | 1.professional  2.skilled workers  3.farmer/farmer’s wife  4.unskilled workers |
| Paternal occupation based on the birth, child welfare and school records. A combined score from these was made. | 1. professional 2. junior clericals   workers | Seven response categories were conducted based on the question concerning paternal occupation:   1. no occupation 2. upper white collar 3. lower white collar 4. skilled worker 5. unskilled worker 6. farmer, field area ≥8 ha 7. farmer, field area <8 ha | 1. professional – upper and lower white-collar  2. skilled worker  3. farmer  4. unskilled worker  5. no occupation | Ten response categories concerning paternal occupation:  1. unskilled or apprentice  2. skilled manual  3.skilled non-manual  4. professional  5. entrepreneur  6. farmer  7. student  8.at home  9. sick pension  10. unemployed | 1.professional  2.skilled workers  3.farmers  4.unskilled workers |
|  |  | Does the family have:  1.an apartment or house of their own  2.a car  Does the family’s dwelling have:  1. electricity  2. telephone  3. running water  4. television  Response options were yes or no |  | Does the family possess:  1.an owner-occupied dwelling  2.a summer cottage  3. a car  4.an automatic washing machine  5. a telephone  6.a central heating  7. a flushing toilet  8 a separate bath room |  |
| Number of people per room - continuous | Number of people per room |  |  |  |  |
| At the age of 41-51 years in HBCS | | At the age of 46-years in NFBC1966 | |  | |
| Higher education based on the register | Higher education  1.university  2. vocational  3 no higher education | Basic education:   1. less than 9 years of basic school 2. basic school 3. matriculation examination   Further education:   1. no occupational education 2. vocational training course 3. vocational school 4. post-secondary education 5. polytechnic education 6. university degree 7. some other education, what? 8. education is unfinished | Basic:  1. matriculation examination  2. no matriculation (basic school or  less)  Higher:  1. university degree – and unfinished education, polytechnic education  2. vocational training - post-secondary education, vocational training course or vocational school  3. no higher education | - | - |
| Occupation based on the register (highest achieves SES) | Occupation   1. High officials 2. Low Officials 3. Self-employed   Labourers | Nine response categories were conducted based on the national register concerning occupation.   1. farmer entrepreneurs 2. entrepreneurs 3. upper white-collar 4. lower white collar 5. workers 6. students 7. pensioners 8. unemployed 9. others | 1. professional – entrepreneurs,  upper and lower white collar  2. manual worker/farmer  3. not currently working | - | - |
|  |  | Participants were asked to select an option to best describe their present employment situation (if along with mainwork you are doing other job or you are studying mark both):   1. permanentfull-timejob 2. permanent part-time job 3. fixed-term full-time job 4. fixed-term part-time job 5. entrepreneur full-time 6. entrepreneur part-time 7. student full-time 8. student part-time 9. unemployed less than ½ years 10. unemployed ½-1 year 11. unemployed over 1 year 12. education or work with labor policy support 13. laid off temporarily or reduced working hours 14. maternity/paternal leave, child care leave 15. retired 16. take care of own household 17. do else, what? | 1. employed – permanent, fixed-term, part-time, entrepreneur  2. not in labour market – retired, student, parental leave, education or work with labor policy support  3. unemployed – orother. | - | - |
| Household income based on the register- continuous | quintiles | - | - | - | - |
|  |  | Do you live:   1. in an owner-occupied flat 2. in a rentalflat 3. in a partially owned flat 4. in a company-owned dwelling 5. in a student dorm 6. supported housing | 1. home ownership (in an owner-occupied flat)  2. no home ownership (in a rental flat, partially owned, company-owned, student dorm, supported housing) | - | - |

Text1. Genotype quality controlfor NFBC1966 and more detailed information concerning calculation of polygenic risk score for body mass index (BMI).

NFBC1966 genotype quality control

Genotyping of the NFBC1966 participants was carried out at the Broad Institute using the Illumina Infinium 370cnvDuo array and the Beadstudio calling algorithm, as described previously by Sabatti*et al.*([1](#_ENREF_1))*.* Individuals were excluded due to call rate < 95%, unspecified sex, sample duplication/contamination, sex mismatch, relatedness (identity by descent [IBD]), outlying heterozygosity or withdrawal of consent, giving a sample size of 5400. Population stratification was assessed by multidimensional scaling analysis (MDS) and compared with Hapmap phase 3 reference populations; no individuals of non-European ancestry were detected. Copy number variations (CNVs) and single nucleotide polymorphisms (SNPs) with call rate < 95% (for markers with minor allele frequency [MAF] > 5%), CNVs and SNPs with call rate < 99% (for markers with MAF <5%), lack of Hardy-Weinberg equilibrium (HWE) (*P*< 1.0 x 10^-4^) or MAF < 1% were excluded. Array genotypes were harmonised and imputed to the Haplotype Reference Consortium (HRC) imputation reference panel ([2](#_ENREF_2)) via the Michigan imputation server ([3](#_ENREF_3)). We excluded imputed SNPs due to minor allele count (MAC) < 5, imputation quality score (*r*^2^) < 0.3 or evidence for Hardy-Weinberg disequilibrium (*P*< 1e-6); we included only autosomal SNPs and used hard called genotypes (as output by the minimac3 software package ([3](#_ENREF_3))) in subsequent analyses.

For calculating for polygenic risk score for BMI BOLT-LMM-model was used.The BOLT-LMM model is similar to the best linear unbiased predictor (BLUP) model, in which SNPs are fitted as random effects in order to account for linkage disequilibrium (correlation between SNPs). Whereas the BLUP model assumes that SNP effects are normally distributed, BOLT-LMM specifies a mixture of Gaussians prior on SNP effects, which accounts for larger-effect SNPs and may yield a PRS which explains a greater proportion of phenotypic variance (4). We estimated BOLT-LMM SNP effects in the UK Biobank (UKB), a prospective cohort of 502,628 volunteers recruited across the UK at age 40–69 years through United Kingdom National Health Service registers (5,6).Participants attended dedicated assessment centres across the UK between 2006 and 2010, during which weight and height were measured by trained study personnel.  We included 457,822 individuals with genotype and BMI data available and self-reported white ethnicity (UKB field ID f.21000.0.0), and fitted the model using 672,345 genotyped autosomal SNPs with MAF > 0.01% and missingness < 10%. We treated the BMI phenotype (f.21001.0.0) similarly to recent genome-wide association studies (4,7): we calculated residuals for BMI regressed on age (f.21003.0.0), age squared, study centre (f.54.0.0) and genotype batch (f.22000.0.0) for men and women separately, which we subsequently normalised using an inverse-normal transformation. We included 20 ancestry informative principal components (PCs) as fixed effects when fitting the BOLT-LMM Bayesian BLUP model, calculated using the “--pca approx” command in plink 2.0 (8) with the same set of SNPs used for fitting the model as described above.

1.Sabatti C, Hartikainen A-L, Pouta A, Ripatti S, Brodsky J, Jones CG, et al. Genome-wide association analysis of metabolic traits in a birth cohort from a founder population. Nature genetics. 2009;41(1):35-46.

2.McCarthy S, Das S, Kretzschmar W, Delaneau O, Wood AR, Teumer A, et al. A reference panel of 64,976 haplotypes for genotype imputation. Nature genetics. 2016;48(10):1279.

3.Das S, Forer L, Schönherr S, Sidore C, Locke AE, Kwong A, et al. Next-generation genotype imputation service and methods. Nature genetics. 2016;48(10):1284.

4. Loh P-R, Kichaev G, Gazal S, Schoech AP, Price AL. Mixed-model association for biobankscaledatasets. Nature genetics. 2018;50:906-908.

5. Bycroft C, Freeman C, Petkova D, Band G, Elliott LT, Sharp K, Motyer A, Vukcevic D,Delaneau O, O'Connell J, et al. Genome-wide genetic data on~ 500,000 UK Biobank participants.BioRxiv. 2017:166298. doi: <https://doi.org/10.1101/166298>

6. Sudlow C, Gallacher J, Allen N, Beral V, Burton P, Danesh J, Downey P, Elliot P, Green J,Landray M et al. UK Biobank: An Open Access Resource for Identifying the Causes of a WideRange of Complex Diseases of Middle and Old Age. PLoS Med 2015;12.

7. Locke AE, Kahali B, Berndt SI, Justice AE, Pers TH, Day FR, Powell C, Vedantam S,Buchkovich ML, Yang J, et al. Genetic studies of body mass index yield new insights for obesitybiology. Nature. 2015;518:197-206.

8. Chang CC, Chow CC, Tellier LC, Vattikuti S, Purcell SM, Lee JJ. Second-generation PLINK: rising to the challenge of larger and richer datasets. Gigascience 2015;4:7.

Table 3. Association of early social disadvantage with visceral fat area (cm^2^),waist circumference (cm) and body fat (%)at 46-years in Northern Finland Birth Cohort 1966 (NFBC1966).

|  | Early social disadvantage | | |  |
| --- | --- | --- | --- | --- |
|  | High | Intermediate | Low |  |
|  | n (%) | n (%) | n (%) |  |
|  | 1463 (25%) | 2900 (50%) | 1444 (25%) |  |
|  | n mean (sd) | n mean (sd) | n mean (sd) | p-value |
| Visceral fat area | 1441 109.58 (43.04) | 2853105.06 (41.10) | 1412 100.78 (40.52) | <0.001 |
| Waist |  |  |  |  |
| Men | 61597.90 (11.97) | 1244 97.61 (11.69) | 675 97.25 (12.09) | 0.613 |
| Women | 840 88.39 (13.74) | 1635 87.25 (13.04) | 758 85.69 (12.30) | 0.0002 |
| Body fat |  |  |  |  |
| Men | 61023.84 (7.48) | 1229 23.27 (6.95) | 664 23.13 (7.25) | 0.164 |
| Women | 83134.20 (8.42) | 1624 33.16 (8.42) | 748 32.12 (8.17) | <0.001 |

Table4. Association between early social disadvantage with visceral fat area (cm^2^) at age of 46-years in Northern Finland Birth Cohort 1966 (NFBC1966, n=3294). Low social disadvantage was set as a reference group.

|  | Visceral fat area | | | |
| --- | --- | --- | --- | --- |
| Early social disadvantage | Estimate (Beta, 95% CI) | | p-value | R^2^ |
| Model 1 |  |  | |  |
| Low | Ref. |  | |  |
| Intermediate | 6.02 (2.55, 9.48) | <0.001 | |  |
| High | 9.47 (5.39, 13.54) | <0.001 | | 0.007 |
| Model 2 |  |  | |  |
| Low | Ref. |  | |  |
| Intermediate | 5.42 (2.09, 8.74) | 0.0014 | |  |
| High | 8.47 (4.49, 12.46) | <0.001 | | 0.10 |
| Model 3 |  |  | |  |
| Low | Ref. |  | |  |
| Intermediate | 4.83 (1.52, 8.13) | 0.004 | |  |
| High | 7.31 (3.33, 11.29) | <0.001 | | 0.11 |
| Model4 |  |  | |  |
| Low | Ref. |  | |  |
| Intermediate | 4.43 (1.15, 7.71) | 0.008 | |  |
| High | 6.54 (2.58, 10.49) | <0.001 | | 0.13 |

Model 1 unadjusted.

Model 2 adjusted for PRS BMI and population stratification (PS).

Model 3 adjusted for PRS BMI, PS and maternal BMI.

Model 4 adjusted for PRS BMI, PS, maternal BMI and sex.

Table5. Associations between early social disadvantage with waist circumference (cm) and body fat (%) at age of 46-years in men and in women in Northern Finland Birth Cohort 1966 (NFBC1966).Low social disadvantage was set as a reference group.

|  | Men |  |  | Women |  |  |
| --- | --- | --- | --- | --- | --- | --- |
|  | Waist circumference | | | | | |
| Early social adversity | Estimate (Beta, 95% CI) | p-value | R^2^ | Estimate (Beta, 95% CI) | p-value | R^2^ |
| Model 1 | n=1448 |  |  | n=1883 |  |  |
| Low | Ref. |  |  | Ref. |  |  |
| Intermediate | 0.92 (-0.46, 2.31) | 0.190 |  | 1.85 (0.35, 3.57) | 0.016 |  |
| High | 0.97 (-0.72, 2.65) | 0.261 | 0.001 | 2.90 (1.17, 4.62) | 0.001 | 0.006 |
| Model 2 |  |  |  |  |  |  |
| Low | Ref. |  |  | Ref. |  |  |
| Intermediate | 0.65 (-0.69, 1.98) | 0.342 |  | 2.01 (0.57, 3.45) | 0.006 |  |
| High | 0.91 (-0.75, 2.57) | 0.282 | 0.080 | 2.86 (1.17, 4.56) | <0.001 | 0.107 |
| Model 3 |  |  |  |  |  |  |
| Low | Ref. |  |  | Ref. |  |  |
| Intermediate | 0.45 (-0.89, 1.78) | 0.512 |  | 1.88 (0.44, 3.31) | 0.010 |  |
| High | 0.64 (-1.02, 2.29) | 0.449 | 0.091 | 2.49 (0.80, 4.19) | 0.004 | 0.115 |
|  |  |  |  |  |  |  |
|  | Body fat | | | | | |
|  | n=1430 |  |  | n=1864 |  |  |
| Model 1 |  |  |  |  |  |  |
| Low | Ref. |  |  | Ref. |  |  |
| Intermediate | 0.45 (-0.42, 1.31) | 0.309 |  | 1.21 (0.25, 2.18) | 0.01 |  |
| High | 0.68 (-0.37, 1.73) | 0.207 | 0.001 | 2.07 (0.96, 3.18) | <0.001 | 0.007 |
| Model 2 |  |  |  |  |  |  |
| Low | Ref. |  |  | Ref. |  |  |
| Intermediate | 0.26 (-0.59, 1.10) | 0.548 |  | 1.31 (0.38, 2.24) | 0.005 |  |
| High | 0.51 (-0.54, 1.56) | 0.340 | 0.060 | 1.99 (0.91, 3.09) | <0.001 | 0.105 |
| Model 3 |  |  |  |  |  |  |
| Low | Ref. |  |  | Ref. |  |  |
| Intermediate | 0.16 (-0.69, 1.00) | 0.715 |  | 1.24 (0.31, 2.16) | 0.009 |  |
| High | 0.37 (-0.68, 1.41) | 0.489 | 0.067 | 1.81 (0.72, 2.90) | <0.001 | 0.110 |

Model 1 unadjusted.

Model 2 adjusted for PRS BMI and population stratification (PS).

Model 3 adjusted for PRS BMI, PS and maternal BMI.

Table 6. Association of change in social disadvantage during the life-course with visceral fat area (cm^2^) at age of 46-years in Northern Finland Birth Cohort (NFBC1966, n=3293). Increased social disadvantage was set as a reference group.

|  | Visceral fat area | | |
| --- | --- | --- | --- |
| Change in social adversity | Estimate (Beta, 95% CI) | p-value | R^2^ |
| Model 1 |  |  |  |
| Increase | Ref. |  |  |
| Stable | 0.70 (-2.60, 4.00) | 0.676 |  |
| Reduced | -2.63 (-6.50, 1.24) | 0.182 | <0.001 |
| Model 2 |  |  |  |
| Increase | Ref. |  |  |
| Stable | 1.04 (-2.11, 4.18) | 0.518 |  |
| Reduced | -2.43 (-6.14, 1.27) | 0.198 | 0.099 |
| Model 3 |  |  |  |
| Increase | Ref. |  |  |
| Stable | 1.19 (-1.94, 4.31) | 0.456 |  |
| Reduced | -2.50 (-6.18, 1.18) | 0.183 | 0.111 |
| Model 4 |  |  |  |
| Increase | Ref. |  |  |
| Stable | -0.10 (-3.21, 3.01) | 0.950 |  |
| Reduced | -5.02 (-8.72, -1.32) | 0.008 | 0.127 |

Model 1 unadjusted.

Model 2 adjusted for PRS BMI and population stratification (PS).

Model 3 adjusted for PRS BMI, PS and maternal BMI.

Model 4 adjusted for PRS BMI, PS, maternal BMI and sex.

Table 7. Association of change in social disadvantage during the lifecourse with waist circumference (cm) and body fat (%) at age of 46-years in men and women in Northern Finland Birth Cohort 1966 (NFBC1966). Increased social disadvantage was set as a reference group.

|  | Men |  |  | Women |  |  |
| --- | --- | --- | --- | --- | --- | --- |
|  | Waist circumference | | | |  |  |
| Change in social adversity | Estimate (Beta, 95% CI) | p-value | R^2^ | Estimate (Beta, 95% CI) | p-value | R^2^ |
|  | n=1447 |  |  | n=1883 |  |  |
| Model 1 |  |  |  |  |  |  |
| Increase | Ref. |  |  | Ref. |  |  |
| Stable | 0.77 (-0.51, 2.05) | 0.239 |  | -1.58 (-3.05, -0.11) | 0.035 |  |
| Reduced | -0.80 (-2.50, 0.91) | 0.359 | 0.002 | -2.87 (-4.50, -1.24) | <0.001 | 0.006 |
| Model 2 |  |  |  |  |  |  |
| Increase | Ref. |  |  | Ref. |  |  |
| Stable | 0.84 (-0.39, 2.08) | 0.180 |  | -1.22 (-2.62, 0.19) | 0.089 |  |
| Reduced | -0.78 (-2.42, 0.87) | 0.355 | 0.083 | -2.51 (-4.07, -0.94) | 0.002 | 0.106 |
| Model 3 |  |  |  |  |  |  |
| Increase |  |  |  |  |  |  |
| Stable | 0.85 (-0.38, 2.07) | 0.177 |  | -1.16 (-2.55, 0.24) | 0.104 |  |
| Reduced | -0.75 (-2.38, 0.89) | 0.371 | 0.093 | -2.55 (-4.11, -0.99) | 0.001 | 0.116 |
|  | Body fat | | | |  |  |
|  |  |  |  |  |  |  |
|  | n=1429 |  |  | n=1864 |  |  |
| Model 1 |  |  |  |  |  |  |
| Increase | Ref. |  |  | Ref. |  |  |
| Stable | 0.62 (-0.18, 1.42) | 0.127 |  | -0.80 (-1.75, 0.15) | 0.098 |  |
| Reduced | 0.24 (-0.82, 1.30) | 0.652 | 0.002 | -1.43 (-2.47, -0.38) | 0.007 | 0.004 |
| Model 2 |  |  |  |  |  |  |
| Increase | Ref. |  |  | Ref. |  |  |
| Stable | 0.62 (-0.16, 1.40) | 0.117 |  | -0.62 (-1.52, 0.29) | 0.183 |  |
| Reduced | 0.22 (-0.82, 1.25) | 0.683 | 0.062 | -1.27 (-2.27, -0.26) | 0.014 | 0.101 |
| Model 3 |  |  |  |  |  |  |
| Increase | Ref. |  |  | Ref. |  |  |
| Stable | 0.63 (-0.15, 1.40) | 0.114 |  | -0.58 (-1.48, 0.32) | 0.206 |  |
| Reduced | 0.23 (-0.80, 1.26) | 0.659 | 0.069 | -1.29 (-2.29, -0.29) | 0.011 | 0.108 |

Model 1 unadjusted.

Model 2 adjusted for PRS BMI and population stratification (PS).

Model 3 adjusted for PRS BMI, PS and maternal BMI.

Figure 1. Directed acyclic diagram (DAG) for the tested association. We may hypothesise the association between exposure to early social disadvantage to be the result of co-existing pathways. This includes the possible interplay with the child polygenic risk score for BMI that might in part proxy some confounding effects of his/her parents’ BMI.


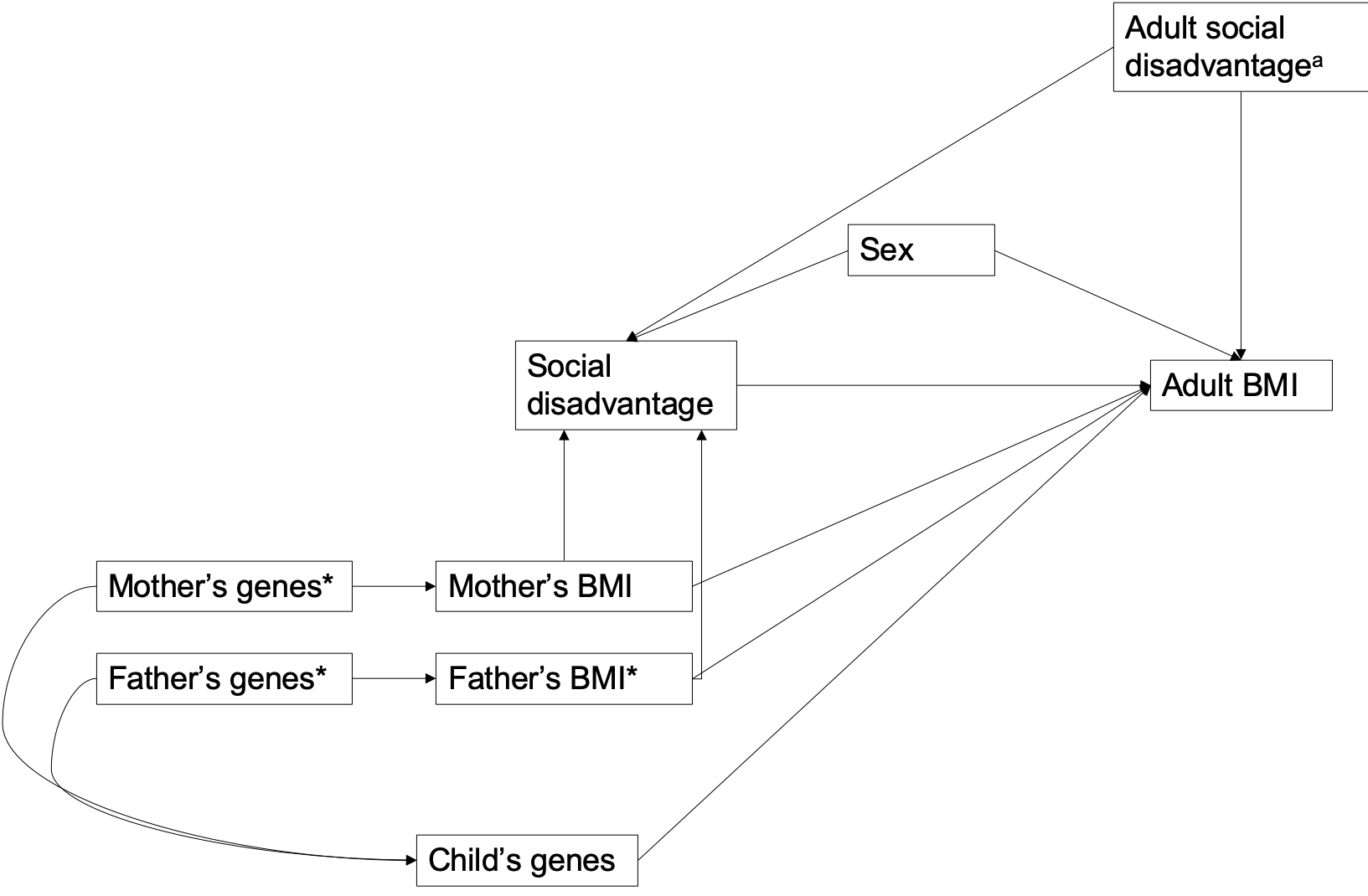

Supplement: Supplementary file 1 — Additional file 1: Table 1A. Variable selection in Helsinki Birth Cohort Study 1934–1944 (✓ included, ✗ excluded). Table 1B. Variable selection in Northern Finland Birth Cohort 1966 (✓ included, ✗ excluded). Table 1C. Variable selection in Northern Finland Birth Cohort 1986 (✓ included, ✗ excluded). Table 2. Social disadvantage variables with their original categorizations and coding used within confirmatory factor analysis in early life in Helsinki Birth Cohort Study 1934–1944 (HBCS1934–1944), Northern Finland Birth Cohort 1966 (NFBC1966), and Northern Finland Birth Cohort Study 1986 (NFBC1986) and at age of 44-years in HBCS1934–1944 and 46-years in NFBC1966. Text 1. Genotype quality control for NFBC1966 and more detailed information concerning calculation of polygenic risk score for body mass index (BMI). Table 3. Association of early social disadvantage with visceral fat area (cm2), waist circumference (cm) and body fat (%) at 46-years in Northern Finland Birth Cohort 1966 (NFBC1966). Table 4. Association between early social disadvantage with visceral fat area (cm2) at age of 46-years in Northern Finland Birth Cohort 1966 (NFBC1966, n = 3294). Low social disadvantage was set as a reference group. Table 5. Associations between early social disadvantage with waist circumference (cm) and body fat (%) at age of 46-years in men and in women in Northern Finland Birth Cohort 1966 (NFBC1966). Low social disadvantage was set as a reference group. Table 6. Association of change in social disadvantage during the life-course with visceral fat area (cm2) at age of 46-years in Northern Finland Birth Cohort (NFBC1966, n = 3293). Increased social disadvantage was set as a reference group. Table 7. Association of change in social disadvantage during the lifecourse with waist circumference (cm) and body fat (%) at age of 46-years in men and women in Northern Finland Birth Cohort 1966 (NFBC1966). Increased social disadvantage was set as a reference group. Figure 1. Directed acycli [file 12889_2020_8763_MOESM1_ESM.docx]
